# Genetic Vectors as a Tool in Association Studies: Definitions and Application for Study of Rheumatoid Arthritis

**DOI:** 10.1155/2015/256818

**Published:** 2015-03-05

**Authors:** Igor Sandalov, Leonid Padyukov

**Affiliations:** ^1^Rheumatology Unit, Department of Medicine Solna, Karolinska Institutet, CMM L8:04, 17176 Stockholm, Sweden; ^2^L.V. Kirensky Institute of Physics, Akademgorodok 50, Krasnoyarsk 660036, Russia

## Abstract

To identify putative relations between different genetic factors in the human genome in the development of common complex disease, we mapped the genetic data to an ensemble of spin chains and analysed the data as a quantum system. Each SNP is considered as a spin with three states corresponding to possible genotypes. The combined genotype represents a multispin state, described by the product of individual-spin states. Each person is characterized by a single genetic vector (GV) and individuals with identical GVs comprise the GV group. This consolidation of genotypes into GVs provides integration of multiple genetic variants for a single statistical test and excludes ambiguity of biological interpretation known for allele and haplotype associations. We analyzed two independent cohorts, with 2633 rheumatoid arthritis cases and 2108 healthy controls, and data for 6 SNPs from the HTR2A locus plus shared epitope allele. We found that GVs based on selected markers are highly informative and overlap for 98.3% of the healthy population between two cohorts. Interestingly, some of the GV groups contain either only controls or only cases, thus demonstrating extreme susceptibility or protection features. By using this new approach we confirmed previously detected univariate associations and demonstrated the most efficient selection of SNPs for combined analyses for functional studies.

## 1. Introduction

The amount of data from genetic studies is growing and its structure is becoming more complex due to the rapid development of new genotyping and sequencing techniques. However, current understanding of the correlation between found genotypes and observed phenotypes remains an obstacle for more efficient use of these data in medicine and biology. There are only a few methods for integration of genetic data in a reasonable way for statistical and functional analyses. An important feature of genetic data is that it is not a collection of random variations, but rather a system with intrinsic correlation between genetic variants. Investigations of study populations are often based on the frequencies of alleles rather than genotypes. However, these variants are distributed on two parallel carriers of information, paired sets of human chromosomes, and in most cases both alleles contribute to the phenotype. Traditionally, statistical analysis in genetics is based on several simplified models with limited options for integration of multiple variants in the same analysis. Although in recent years an exponential growth of different approaches to search for epistatic effects, or gene-gene interaction, in studies of complex traits is observed (see review [1]), a simultaneous consideration of multiple variants remains difficult due to “bottom-up” the design of the strategy of the search.

In this study we suggest and evaluate a genetic vector approach (GVA; briefly described in [2]) that would allow integration of available genotyping data in an unambiguous way, selection of the most representative combinations for the statistical analyses, and, finally, significant reduction of the number of variants that need to be inspected for functional studies. Our approach provides possibility to combine the advantage of joining several genetic markers in one combination (haplotype) with consideration to genotype (not allele) for each marker. Instead of ambiguous connection of allele or haplotype to certain phenotype in each individual, in GVA each individual has only a single genetic vector that corresponds to the phenotype. Thus the genotype-phenotype relation is unambiguous and it does not need* a priori* biological interpretation (dominance/recessiveness). GVA also permits expression of the traditional measures of the association for separate genotypes and, particularly, enables detection of gene-gene interaction.

## 2. Methods

### 2.1. Genetic Vectors for Association Studies

Due to the complexity of genetic data, multiple options for the analyses are available. However, the majority of genetic analyses are based on univariate tests for independent genetic markers and applications with simultaneous employment of several genetic markers are relatively rare. Based on linkage disequilibrium, haplotypes are considered to be a valuable tool [3], but this approach does not provide unambiguous assessment to phenotype, since individuals with heterozygotic state could be, at best, considered as an intermediate group or should be combined with homozygotic state in the absence of a biological hypothesis. We constructed an approach for genetic analyses that integrates a number of markers as a whole entity, regardless of the specific linkage on a particular chromosome.

The design of the model is based on integration of different genetic variants in a reasonable biological group by assigning each individual to a sequence of genotypes in relation to given genetic variants. Since the combinations available in the population are not random and reflect the population's genetic structure, the number of these sequences of genotypes is significantly less than the number of individuals in a study population of reasonable size. The individuals identical in the order of genotypes represent a group, which may have differential representation of phenotypes. In a case-control design the frequencies of these groups could be statistically compared to identify those that are most likely to associate with a selected phenotype.

Imagine that we are interested in the degree of association to a certain phenotype of several single-nucleotide polymorphisms (SNPs), which may belong to the same, or to different chromosomes. A polymorphism is defined at the level of the whole study population, whereas for a particular person each SNP has a definite genotype value. Let us make the following convention: we choose and fix the order of the SNPs. Then each person will be characterized by the set of genotypes that can be thought of as a* genetic vector* (GV), characterizing a particular person. The number of genetic variations included in this vector is the length of the GV. The study population is then characterized by the set of GVs.

In an association study, each GV *ν*_*i*_ “contains” some number *n*_*h*,*i*_ of controls and *n*_*s*,*i*_ cases. Then the structure of the study population is described by the Table 1.

Thus, this is a simple way to avoid working with separate alleles, and even with genotypes; instead, the focus lies on their relevant combinations in the study population, that is, on the genetic vectors. One of the advantages worth mentioning is that if there exists some, say, genotype-genotype interactions, these will be taken into account automatically, since at this first stage the approach does not require any assumptions about the statistical nature of the GV's constituents: this is simply one of the possible ways to represent the experimental data set. Since we now know the number of healthy controls and number of cases, as well as the total numbers of both, we can apply standard statistical machinery, testing the statistical hypotheses for whole GVs; particularly, in order to discover the “most promising” GVs, we can (and will) evaluate the odds ratios, the regions with the greatest fluctuations on the basis of, say, Fisher's exact tests, and so on.

The GVA consists of several stages:

1. selection of genetic markers,
2. genotyping for two study populations,
3. assigning of genetic vector value to each individual,
4. sorting all individuals in the study population into groups, according to particular GVs and chosen phenotype parameters,
5. exclusion of GVs with zero number of individuals in any subgroup (subset),
6. computation of odds ratio and relative risk (OR/RR), pathogenic, and protective genetic vectors,
7. sorting GVs according to statistical confidence,
8. exclusion of GVs with low confidence,
9. analysis of the match between study populations: focus on replication and absence of opposite effect for the vector.

The mathematical details of the GVA itself are given below (Section 2.3–2.5).

### 2.2. Experimental Data Sets and GV Definitions

To demonstrate the capacity of the methodology we applied it to the analyses of data from two study populations: from the Swedish Epidemiological Investigation of Rheumatoid Arthritis (EIRA, 1820 cases and 947 controls) and from the North American Rheumatoid Arthritis Consortium (NARAC, 813 ACPA-positive cases and 1161 controls).

The EIRA study population is a population-based case-control study of incident cases of rheumatoid arthritis (RA) in which all patients fulfilled the American College of Rheumatology (ACR) 1987 criteria [4]. Controls were randomly selected from the Swedish national population registry, taking into consideration the patient's age, sex, and residential area. More details about the EIRA study population have been described elsewhere [5].

The cases in the NARAC study population consisted of RA patients of self-reported white ancestry, who were randomly drawn from four different groups of patients, while controls were recruited from the New York Cancer Project [6, 7]. Both studies were conducted after obtaining approval from the Regional Ethics Committees and in accordance with the Declaration of Helsinki.

Below, we apply GVA for the analysis of EIRA's and NARAC's association to rheumatoid arthritis (RA) using the data for 6 SNPs from the HTR2A locus, rs6314, rs977003, rs1328674, rs2070037, rs6313, and rs6311 and for HLA-DRB1 shared epitope alleles. We defined SE alleles as any of HLA-DRB1^*^01 (not ^*^0103), ^*^04 and ^*^10; previous study suggested very low frequency for non-SE HLA-DRB1^*^04 variants in Caucasian populations [8]. Conventional evaluation for HTR2A genetic markers was performed previously [9].

We defined the GV states as a product of SNP states, as shown in Table 2. In order to compare the GV structure between the EIRA and NARAC study populations and to perform statistical evaluation, we, first, assigned individuals to a particular GV group using individual genotypes and, second, normalized the number of people, for each GV (according to (10) below).

### 2.3. Mathematical Details of the Genetic Vector Approach (GVA)

Let us consider a population, consisting of *N*_*h*_ controls (*h* = healthy) and *N*_*s*_ (*s* = sick) cases. The number of genetic markers is a matter of preliminary research and will be assigned as *N*_SNP_. We choose here SNPs for simplicity, but the approach can be easily extended for multiallelic markers. There are no restrictions on the position of markers within chromosomes, and variants from different chromosomes could be used. In the whole study population, with the dizygotic human genome, each SNP can normally produce 3 different categorical values, or genotypes. However, these categories are exclusive and only one of the categories can be found in the DNA of an individual person. This is similar to one of the components (say, *z*) of spin *S* = 1, which can acquire three values, *S* ^*z*^ = 1,0, −1, and could be described by corresponding three states, |1〉, |0〉, |−1〉. The description of the study population with a set of SNPs is similar to the description in physics of an ensemble of spin chains (see, e.g, [10]). Each of the spin chains contains *N*_SNP_ spins and can be in different quantum states. The latter is described by the set of vectors:

(1) $$\begin{array}{c} |\nu_{i}\rangle =|S_{1i}\rangle |S_{2i}\rangle |S_{3i}\rangle \cdots |S_{N_{\text{SNP}},i}\rangle |\Upsilon \rangle , \end{array}$$

where *Υ* represents other variables; the spin corresponds to the SNP, and its values correspond to the genotypes. Therefore, a combination of genotypes form a vector for each person with known genotypes and we refer to these combinations as* genetic vectors* (GVs).

Different statistical characteristics of the study population, such as the average frequency of people with a certain genotype and correlations between genotypes and phenotypes, can be obtained by, first, averaging certain spin-operator combinations on these multispin states and, second, averaging the result over the population of interest. However, it is more convenient for us to speculate not in terms of ${\hat{S}}^{z}$-operators, but, rather, in terms of the operators, defined as follows:

(2) $$\begin{array}{c} {\hat{X}}_{\nu i}^{\gamma }=|\nu ,i,\gamma \rangle \langle \nu ,i,\gamma |;\\ {\hat{X}}_{\nu i}^{\gamma }|\nu_{1},i_{1},\gamma_{1}\rangle =\delta_{\nu \nu_{1}}\delta_{ii_{1}}\delta_{\gamma \gamma_{1}}|\nu ,i,\gamma \rangle . \end{array}$$

These variables can be interpreted as the operators of population numbers (PNs) of the *γ*-state in the cell *i* of the genetic vector *ν*. The *X*-operators are projection operators: (*X*_*νi*_^*γ*^)^2^ = *X*_*νi*_^*γ*^, *γ* = 1,2, 3. Notice that the projection operators are sometimes used for description of spin systems, since the spin operators can be easily expressed in terms of *X*_*νi*_^*γ*^-operators. For example, *z*-projecture of spin operator is ${\hat{S}}_{\nu i}^{z}=\sum_{\gamma }m_{\gamma }X_{\nu i}^{\gamma }=1\cdot X_{\nu i}^{1}+0\cdot X_{\nu i}^{2}+\left(-1\right)\cdot X_{\nu i}^{3}=X_{\nu i}^{1}-X_{\nu i}^{3}$; that is, it is just the difference between the population-number operators of two states.

In genetics this multiple-spin state, describing the combination of genotypes, GV, and characterizing each person, could be obtained from the experimental genotyping. Then, one can compare all expected values of interest in groups via the expectation values of corresponding operators on the wave functions (GVs) for two ensembles, for example, controls and cases. We are dealing here with a static problem: all the states of “spin chains” are given; they do not depend on time; the total wave function |Ψ^*α*^〉 of each of the ensembles *α* = ctrl, case is a product of the wave functions of separate “chains,” ∏_*ν*_ | *ν*〉. The expectation value of the population-number operator for a certain person is

(3) Nνiγα≡ΨαXνiγΨα=νXνiγν=1if  i,γ∈GVν;0otherwise.

Therefore, in general, the individual *ν* is characterized by 3*N*_SNP_ numbers (*i* = 1,…, *N*_SNP_, and *γ* = 1,2, 3, if other parameters are excluded) or by the matrix 3 × *N*_SNP_ of PNs for each *ν*. We will refer to it as* index matrix* (IM):

(4) $$\begin{array}{c} {\hat{C}}_{\nu }\equiv \left\|N_{\nu i\gamma }\right\|. \end{array}$$

Thus, an individual is fully characterized by this matrix as described above. Each column, describing the filling of the “spin-one state”, can contain only one nonzero value equal to one and two others equal to zero. Each column represents a particular variation and, for a SNP, has three levels defined by nucleotides at the SNP position. The matrix for selected genotypes is a matter of experimental design and may reflect either a set of selected SNPs of interest or a sequence of all SNPs at a certain locus. For example, if we are interested in an ensemble of three SNP chains, with SNP_1_ = *A*, SNP_2_ = *B*, and SNP_3_ = *C*, with *A* = *a*_1_, *a*_2_, *a*_3_, *B* = *b*_1_, *b*_2_, *b*_3_, and *C* = *c*_1_, *c*_2_, *c*_3_, where *a*_*i*_, *b*_*j*_, *c*_*k*_ are corresponding genotypes, then some people may be in the state characterized by the genetic vector:

(5) $$\begin{array}{c} |\nu_{0}\rangle ={|a_{2}\rangle }_{1}{|b_{1}\rangle }_{2}{|c_{3}\rangle }_{3}. \end{array}$$

Corresponding to this GV index matrix is

(6) $$\begin{array}{c} C_{\nu_{0}}=\left(\begin{array}{ccc} 0 & 1 & 0\\ 1 & 0 & 0\\ 0 & 0 & 1 \end{array}\right). \end{array}$$

These IMs will be used in our further calculations. For statistical evaluation of differences between controls and cases we will develop two matrices 3 × *N*_SNP_ of PNs:

(7)   Niγα=1Nα∑νΨαXνiγΨα,α=h  i.e.,ctrl,  s  i.e.,case.

These, in fact, are the genotype-frequency maps for controls and cases for the study population of interest.

In the same fashion we can obtain the rate for people, who have a pair of certain genotypes (*i*, *γ*) and (*i*′, *γ*′); this rate can be calculated from the expression:

(8) $$\begin{array}{c} P_{i,\gamma ;i^{\prime },\gamma^{\prime }}^{\alpha }=\frac{1}{N_{\alpha }}\underset{\nu }{\sum }\langle \Psi^{\alpha }|X_{\nu i}^{\gamma }X_{\nu i^{\prime }}^{\gamma^{\prime }}|\Psi^{\alpha }\rangle ,\;\alpha =h,s. \end{array}$$

The expression for triplets contains a product of three *X*-operators and so on.

Each GV *ν*_*i*_ “contains” some number *n*_*h*,*i*_ of controls and *n*_*s*,*i*_ cases, whereas the structure of the study population is described by Table 1.

Notice that the set of GVs is orthonormalized

(9) $$\begin{array}{c} \langle \nu_{i}\;|\;\nu_{j}\rangle =\delta_{ij}=\left\{\begin{array}{cc} 1, & i=j;\\ 0, & i\neq j \end{array}\right. \end{array}$$

and, therefore, can be considered as a basis set in *N*_GV_-dimensional space, whereas each part of the study population, controls and cases, can be presented as a point in this space. If some other study population contains GVs that are not included in the set |*ν*_1_〉, |*ν*_2_〉,…, |*ν*_*N*_GV__〉, the set should be complemented by these GVs in order to obtain a complete set for the extended population, including all populations in question.

### 2.4. Statistical Treatment of GVs

In order to avoid possible confusion it is important to remember that each GV describes the set of SNP values. If the population has a group of individuals with the same GV, we can use this fact in the statistical treatment of the population, describing the whole population in terms of these larger-scale variables, GVs. Then, each GV within a given study population is characterized by the number of healthy controls *N*_*h*_ and the number of cases *N*_*s*_ and we can introduce two frequencies:

(10) $$\begin{array}{c} p_{\nu }^{\alpha }=\frac{n_{\nu }^{\alpha }}{N_{\alpha }},\;\alpha =h,s. \end{array}$$

Comparing these, we can determine which of the GVs are more protective and which are more pathogenic. As a criterion for the separation of GV subgroups with protective and pathogenic behavior we choose here the odds ratio, defined by GV frequencies:

(11) $$\begin{array}{c} {\text{OR}}_{\nu }^{\left(\text{GV}\right)}=\frac{p_{\nu }^{s}/\left(1-p_{\nu }^{s}\right)}{p_{\nu }^{h}/\left(1-p_{\nu }^{h}\right)}. \end{array}$$

The difference between the standard definitions of OR and the one used here is discussed in the book [10, page 154]. Further, we have to choose some threshold OR_tr⁡_, which reflects a chosen criterion of statistical confidence, and select all GVs that satisfy the condition

(12) $$\begin{array}{c} {\text{OR}}_{\nu }^{\left(\text{GV}\right)}>{\text{OR}}_{\mathrm{tr},\nu }^{s} \end{array}$$

for “pathogenic” GVs and for the “protective” ones, for which OR_*ν*_^(GV)^ < OR_tr⁡,*ν*_^*h*^. The standard obvious threshold in (12) would be OR_tr⁡,*ν*_ = OR_tr⁡,*ν*_^*h*^ = OR_tr⁡,*ν*_^*s*^ = 1. However, statistical fluctuations in the vicinity of the threshold OR_tr⁡,*ν*_ = 1 may make the choice of the groups, described by certain GVs, doubtful. In order to garantee that the selected GVs remain on a safe side, one can choose a harder criteria of the GV selection. Of course, the threshold for “protective” GVs OR_tr⁡,*ν*_^*h*^ not obligatory should be chosen equal to 1/OR_tr⁡,*ν*_^*s*^. Due to the different number of people in each GV, the threshold value OR_tr⁡,*ν*_ can occur as *ν*-dependent. Let us denote the subset of GVs defined by the inequality (12) as *Ω*_trust_ (the details of this subset will be discussed in Sections 2.5 and 3.2). Then the combination of variants, responsible for a large OR, can be visualized by summing the IMs of the GVs that fulfil (12). This summation produces two matrices,

(13) $$\begin{array}{c} \bar{C_{\Omega_{\text{crit}}}^{h}}=\frac{1}{N_{h}}\underset{\nu }{\sum }C_{v,\Omega_{\text{trust}}}^{h},\;\;\bar{C_{\Omega_{\text{crit}}}^{s}}=\frac{1}{N_{s}}\underset{\nu }{\sum }C_{v,\Omega_{\text{trust}}}^{s}, \end{array}$$

which represent frequencies of genotypes in selected control and case groups. The cells in the matrix $\Delta \bar{C_{\Omega_{\text{trust}}}}=\bar{C_{\Omega_{\text{trust}}}^{s}}-\bar{C_{\Omega_{\text{trust}}}^{h}}$ that contain the largest difference give us a possible indication of which of the genotypes, entering different GVs, are responsible for the large ORs displayed by the GVs.

### 2.5. The Choice of Thresholds

Let us now discuss a criterion for the choice of thresholds OR_tr⁡,*ν*_. In the first step, all *N*_*t*_ individuals in the population are sorted in terms of corresponding genetic vectors (GV) of a length *N*_GV_. Each GV *ν* contains *n*_*ν*_^*h*^ of healthy controls (HCs) and *n*_*ν*_^*s*^ cases, with a total of *n*_*ν*_^*t*^ = *n*_*ν*_^ctrl^ + *n*_*ν*_^case^ individuals in each GV. The whole population consists of *N*_*h*_ controls and *N*_*s*_ case individuals, with the total number of individuals in the population *N*_*t*_ = *N*_*h*_ + *N*_*s*_. Then for each GV *ν* we can write down the contingency Table 3 and the odds ratio

(14) $$\begin{array}{c} {\text{OR}}_{\nu }\left(n_{\nu }^{s}\right)=\frac{n_{\nu }^{s}\left(N_{h}-n_{\nu }^{h}\right)}{n_{\nu }^{h}\left(N_{s}-n_{\nu }^{s}\right)}, \end{array}$$

based on this table. The full set of GVs can be sorted into two subsets, one with OR > 1, which reflects the pathogenic combinations, and the rest with OR < 1, corresponding to the protective combinations of genotypes.

At this stage we have to estimate the degree of confidence for each OR_*ν*_. In order to do this, we introduce the random variable *n*_*νk*_, which describes a number of case individuals, selected randomly from *n*_*ν*_^*t*^ persons, with the number *n*_*ν*_^*t*^ *kept fixed*. Then the probability to pick up precisely this number for fixed values *n*_*ν*_^*t*^, *N*_*h*_, and *N*_*s*_ is described by the hypergeometric distribution (as used in Fisher's exact test):

(15) $$\begin{array}{c} \frac{\left(\begin{array}{c} N_{s}\\ n_{\nu k}^{s} \end{array}\right)\left(\begin{array}{c} N_{h}\\ n_{\nu k}^{h} \end{array}\right)}{\left(\begin{array}{c} N_{t}\\ n_{\nu }^{t} \end{array}\right)},\;n_{\nu k}=0,1,2,\ldots ,n_{\nu }^{t}, \end{array}$$

where $\left(\begin{array}{c} N\\ n \end{array}\right)$ are binomial coefficients. Constructing the complex random variable of interest, OR, for the chosen *n*_*νk*_,

(16) $$\begin{array}{c} {\text{OR}}_{\nu k}^{\text{rand}}=\frac{n_{\nu k}\left(N_{h}-n_{\nu }^{t}+n_{\nu k}\right)}{\left(n_{\nu }^{t}-n_{\nu k}\right)\left(N_{s}-n_{\nu k}\right)}, \end{array}$$

we can calculate for each GV the average odds ratio

(17) $$\begin{array}{c} \mu_{1}\left(\text{OR}\right)=M\left[{\text{OR}}_{\nu k}^{\text{rand}}\right]=\overset{n_{\nu }^{t}-1}{\underset{k=1}{\sum }}\frac{n_{\nu k}\left(N_{h}-n_{\nu }^{t}+n_{\nu k}\right)}{\left(n_{\nu }^{t}-n_{\nu k}\right)\left(N_{s}-n_{\nu k}\right)}p\left(n_{\nu k}\right), \end{array}$$

and, say, variance, *μ*_2_(OR) = var[OR_*νk*_^rand^] = *M*[(OR_*νk*_^rand^)^2^] − (*M*[OR_*νk*_^rand^])^2^. This would be sufficient if we had normal distribution of the variable of interest, OR_*νk*_^rand^, but our case is far from this. One could also try to use the higher moments in order to build the confidence intervals for OR_*ν*_^rand^ for each GV *ν*. However, due to asymmetry of the distribution for the odds ratio OR_*νk*_^rand^ and the presence of GVs with a small total number *n*_*ν*_^*t*^ of individuals in GV, this method is impractical. Instead, we build the distributions for the complex random variable OR_*νk*_^rand^, *p*(OR_*νk*_^rand^), and find the thresholds (OR_lower_^tr⁡^, OR_upper_^tr⁡^) for the interval, where OR_*νk*_^rand^ experiences the largest fluctuations (for the normal distribution this would be the interval [−*ησ*, *ησ*], where *σ* is the standard deviation and $\left[1-\mathrm{erf}(\eta /\sqrt{2})\right]/2$ is chosen accuracy) directly from the equation based on this distribution:

(18) $$\begin{array}{c} \alpha_{\nu ,\text{upper}}=\underset{{\text{OR}}_{\nu k}^{\text{rand}}>{\text{OR}}_{\text{upper}}^{\mathrm{tr}}}{\sum }p\left({\text{OR}}_{\nu k}^{\text{rand}}\right),\\ \alpha_{\nu ,\text{lower}}=\underset{{\text{OR}}_{\nu k}^{\text{rand}}<{\text{OR}}_{\text{lower}}^{\mathrm{tr}}}{\sum }p\left({\text{OR}}_{\nu k}^{\text{rand}}\right). \end{array}$$

We choose a 5% level of accuracy, that is, *α*_*ν*,upper/lower_ = 0.05. Since the full number of steps in the discrete distribution *p*(*n*_*νk*_; *n*_*ν*_^*t*^, *N*_*s*_, *N*_*h*_) for the total number of individuals in GV *n*_*ν*_^*t*^ is restricted by *n*_*ν*_^*t*^, the best accuracy *α* that can be achieved is also restricted by the magnitude of this step; the sum in (18) will consist of only one term with this minimal possible step. As normal, a decrease of *α* also decreases the number of contributing GVs, decreasing the total statistical power.

The other way to estimate the degree of confidence for the experimentally found OR_*ν*,exp⁡_ for each GV is to evaluate the sum of probabilities for all possible OR_*νk*_^rand^ exceeding OR_*ν*,exp⁡_:

(19) $$\begin{array}{c} P_{\nu ,\text{CI}}=\underset{{\text{OR}}_{\nu k}^{\text{rand}}>{\text{OR}}_{\nu ,\exp}}{\sum }p\left({\text{OR}}_{\nu k}^{\text{rand}}\right). \end{array}$$

**Figure 1:**
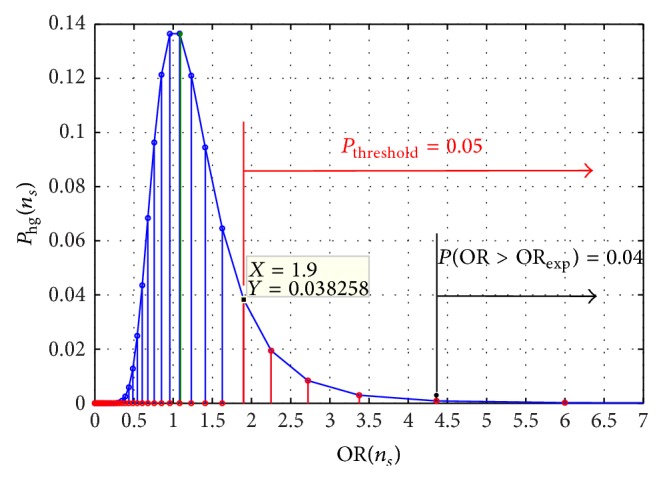
The example of the OR-distribution at fixed *n*_*ν*_^*t*^: the density of probability for hypergeometric distribution *P*_*hg*_(*n*_*s*_) versus odds ratio OR(*n*_*s*_). As seen, the distribution is asymmetric. The plot is built for the following parameters: *N*_*s*_ = 1500; *N*_*h*_ = 779; *n*_*ν*_^*t*^ = 37. The expectation value *μ*_1_(OR_*νk*_^rand^) = 1.0929 (shown by green line), the threshold OR, OR_upper_^tr⁡^ = 1.9. This is the solution to (18), meaning that the sum of probabilities from *P*(OR_upper_^tr⁡^) till *P*(OR_max⁡_) (shown by red vertical lines) is equal to *α* = 0.05. At the end, the probability of finding OR greater than the experimental value of the odds ratio, OR_*ν*,exp⁡_ = 4.3584, equals 0.04; the value OR_*ν*,exp⁡_ is taken from experiments, described above, and is shown by a black star (∗).

The criteria are illustrated in Figure 1.

We use both criteria.

The GVs, which contain either only cases (*n*_*ν*_^*h*^ = 0, *n*_*ν*_^*s*^ ≠ 0,* i.e.*, “solely sick”) or only controls (*n*_*ν*_^*h*^ ≠ 0, *n*_*ν*_^*s*^ = 0,* i.e*., “solely healthy”), require separate consideration. For simplicity we call them “zero GVs.”

## 3. Results and Discussion

### 3.1. GV Frequency Distributions

**Figure 2:**
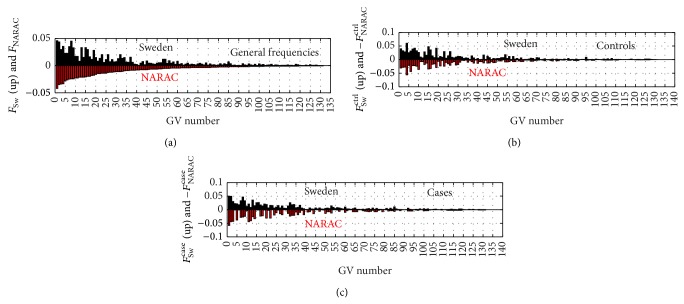
Comparison of frequencies of GVs for EIRA (black bars) and NARAC (red bars). All GVs are ordered with respect to general frequencies of NARAC. Frequencies in (a) are defined as *F*_coh_ = (*n*_*H*_^(coh)^ + *n*_*I*_^(coh)^)/*N*_tot_^(coh)^, coh = EIRA, NARAC.

We have found that all individuals in two study populations, based on selected SNPs from HTR2A and HLA-DRB1 SE alleles for the chosen GV length (number of markers), can be ascribed to 161 GVs for EIRA and 163 GVs for NARAC; 131 of them are common GVs for both studies. The total basis of GVs for these both cohorts, therefore, consists of 193 GVs, while Figure 2 displays the results for common GVs only.

The rest of the GVs contain a relatively small number of individuals in each of the cohorts: 40 people of 2767 total in 30 GVs (1.45%) in EIRA and 36 people of 1974 total in 32 GVs (1.82%) in NARAC. Since each of these noncommon GVs contains just one or two people, it is obvious that these relatively rare GVs would be unlikely to give a significant contribution to the statistical characteristics of the cohorts. Thus, the vast majority of GVs in both cohorts are the same and it is reasonable to compare GV frequencies between the two study populations.

The data in Figure 2 demonstrate the high level of coherency of the GV distribution within each study population (EIRA and NARAC) and provides a rationale for statistical comparison between RA cases and controls. It is also evident that these two populations differ in the frequencies of specific GVs, which is likely to reflect a different genetic backgrounds or differences in the selection criteria. More specifically, as can be seen from Figure 2(c), frequencies of several GVs for the RA cases in the two studies differ significantly, with some GVs absent from NARAC, while relatively common in EIRA.

### 3.2. Stratification of the Common GVs and an Analysis of the Consistency

In order to facilitate identification and comparison between high-risk and protective GVs in the two study populations, we selected GVs which display OR > OR_upper_^thr^ or OR < OR_lower_^thr^ (see (18) in Section 2.5). However, the GVs with either *n*_*ν*_^ctrl^ = 0 or *n*_*ν*_^case^ = 0 should be considered separately, since in this case OR cannot be formally defined. These subgroups of GVs with zero values will be described in Section 3.4. The distribution of GVs between the groups is shown in Table 4.

After exclusion of the GVs specific only for one study and those with zero frequency in any of subsets, we analysed the set of GVs with corresponding numbers of individuals, as shown in Table 5.

**Figure 3:**
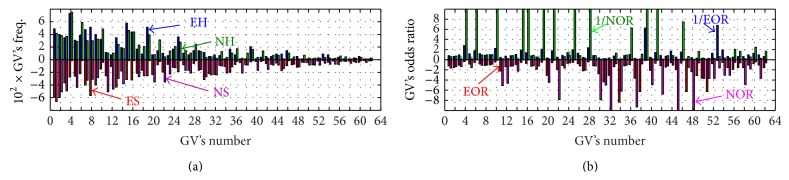
Comparison of the data for EIRA and NARAC subsets of GVs that do not contain either *n* ^*h*^ = 0 or *n* ^*s*^ = 0. All GVs are sorted with respect to frequencies *f*_*ν*,NARAC_^tot^ = (*n*_*ν*,NARAC_^*h*^ + *n*_*ν*,NARAC_^*s*^)/∑_*ν*_(*n*_*ν*,NARAC_^*h*^ + *n*_*ν*,NARAC_^*s*^); (a) displays frequencies of GVs: *f*_EIRA_^*h*^ (blue), *f*_NARAC_^*h*^ (green), -*f*_EIRA_^*s*^ (red), -*f*_NARAC_^*s*^ (lilac); the case-imaging with minus sign is used for a better visibility; EH,ES and NH,NS denote the frequencies of EIRA′ (E) and NARAC′(N) GVs for healthy (H) controls and cases (S). (b) Odds ratios for GVs, the color notations are the same as for frequencies, and the notations are 1/EOR ≡ OR^EIRA^(*h*/*s*), 1/NOR ≡ OR^NARAC^(*h*/*s*), −EOR ≡ −OR^EIRA^(*s*/*h*), −NOR ≡ −OR^NARAC^(*s*/*h*). There are several GVs that demonstrate OR > 9.

As seen from Table 5, despite the fact that the number of GVs decreased more than twice after exclusion of nonoverlapping GVs and GVs with zero values, the remaining GVs represent 76–88% of individuals. The data for the set of common GVs with both *n* ^*h*^ ≠ 0 and *n* ^*s*^ ≠ 0 are displayed in Figure 3.

In order to estimate quantitatively the degree of consistency of the data, we stratified the set of common GVs into four categories using OR, defined by (11):

(20) $$\begin{array}{c} \Omega_{11}:\left\{\text{O}{\text{R}}^{\text{EIRA}}>1\;\;\&\;\;\text{O}{\text{R}}^{\text{NARAC}}>1\right\};\\ \Omega_{12}:\left\{\text{O}{\text{R}}^{\text{EIRA}}>1\;\;\&\;\;\text{O}{\text{R}}^{\text{NARAC}}<1\right\};\\ \Omega_{21}:\left\{\text{O}{\text{R}}^{\text{EIRA}}<1\;\;\&\;\;\text{O}{\text{R}}^{\text{NARAC}}>1\right\};\\ \Omega_{22}:\left\{\text{O}{\text{R}}^{\text{EIRA}}<1\;\;\&\;\;\text{O}{\text{R}}^{\text{NARAC}}<1\right\}. \end{array}$$

**Figure 4:**
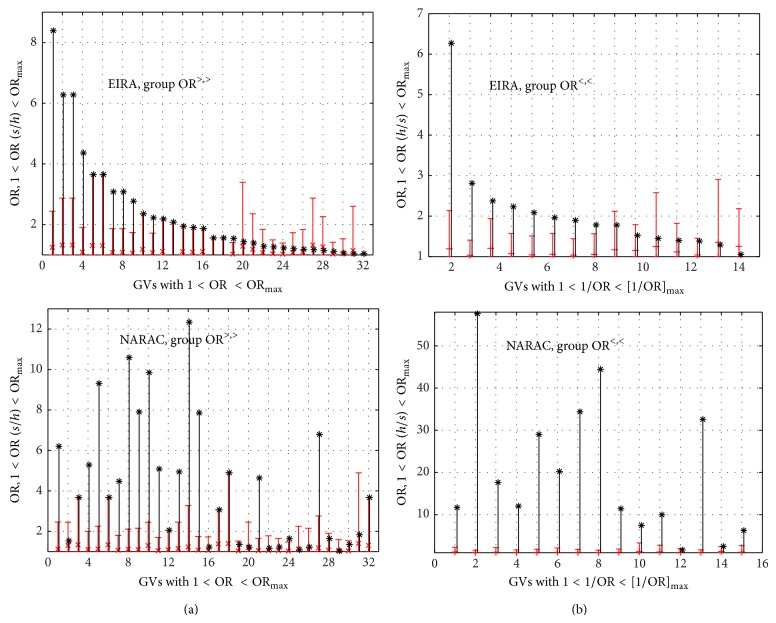
Odds ratios for EIRA and NARAC for common GVs “without zeros” (*n*_*s*_ ≠ 0 and *n*_*h*_ ≠ 0). The red stars represent the expectation value 〈OR〉 of random OR (notice that 〈OR〉 is slightly different from unity due to asymmetry and discreteness of the distribution for OR); error bars show the interval that is hit by OR with randomly chosen *n*_*ν*_^*s*^ from the interval (0,1, 2,…, *n*_*ν*_^*t*^ − 1) with 95% probability; black stars display observed OR for given GV. Thus, if OR_*ν*,exp⁡_ is above the upper error bar, the null hypothesis that the observed value of OR can arise occasionally is not supported with 5% threshold value (for the random variable *n*_*s*_ = 0,1, 2,…, *n*_*t*_ − 1).

The result of this is summarized in Table 6 and only GVs with consistency in direction of the effect (risk or protectivity) were selected for futher analyses; see Tables 7 and 8. At the next step, using (17) for the average OR for each GV and (18) at the level *α* = 0.05, we can construct the plot for the *Ω*_11_ group of GVs: 〈OR〉, OR_*ν*,upper_^tr⁡^ and the “experimental” value of OR. Subsequently, for the *Ω*_22_ group, where all OR < 1, we used OR^EIRA^(*h*/*s*) = 1/OR_*Ω*_22__^EIRA^(*s*/*h*), OR^NARAC^(*h*/*s*) = 1/OR_*Ω*_22__^NARAC^(*s*/*h*). The results are displayed in Figure 4.

In this comparison, the NARAC group has a much greater size of ORs. However, some of the NARAC GVs happen to be inconsistent with the ones from EIRA. Therefore, only those GVs which exceed the upper 5% threshold* in both* cohorts were chosen for further analysis.

**Figure 5:**
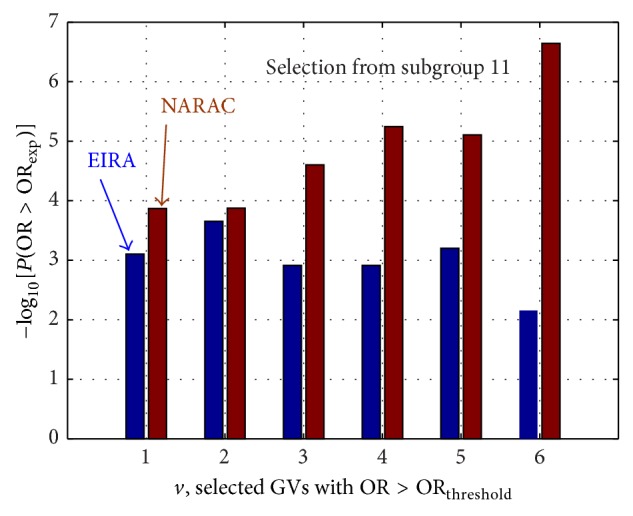
Negative logarithm of the probability to find OR_*ν*_^rand^ > OR_*ν*,exp⁡_ in the subgroup *Ω*_11_ : {OR^EIRA^ > 1  &  OR^NARAC^ > 1}.

We first analyzed the risk groups, *Ω*_11_, represented in the left panels of Figure 4 and found that only 6 GVs, for which OR_*ν*,exp⁡_ > OR_threshold_^right^ in both cohorts, give consistent results. Let us inspect the probabilities of occasionally finding an OR greater than OR_*ν*,exp⁡_ in the selected GVs. The result is presented in Figure 5.

Thus, since these subgroups fulfill both criteria, namely, the GVs display OR beyond the fluctuation width near 〈OR〉 for chosen *α* = 0.05 and show a low probability of finding OR_*ν*_^rand^ > OR_*ν*,exp⁡_; the combination of variants corresponding to this subgroup GVs should be considered as most probable candidates for an association with RA.

**Figure 6:**
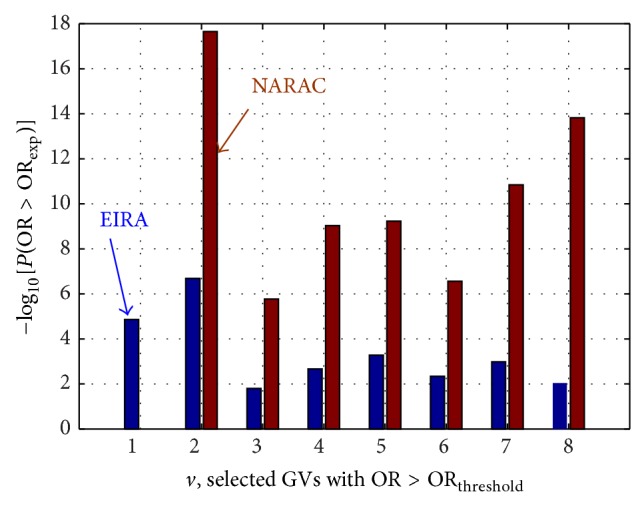
The values of −log⁡_10_ *P*(OR_*ν*_^rand^ < OR_*ν*,exp⁡_) for the consistent GVs in the subgroup *Ω*_22_ : {OR^EIRA^ < 1  &  OR^NARAC^ < 1}.

Among the subgroup of “protective” genotype combinations, which are represented in the right panels of Figure 4, only the first eight GVs give consistent results in both the EIRA and NARAC subgroups. In contrast, the NARAC GVs from 9 to 15, which display OR_exp⁡_ < OR_threshold_^left^, do not fulfil similar requirements in the EIRA subgroup. The values of −log⁡_10_ *P*(OR_*ν*_^rand^ < OR_*ν*,exp⁡_) for the consistent GVs in this subgroup are presented in Figure 6.

The probabilities shown in Figure 6 suggest that a protective role is played by the combinations of variants, presented by these GVs.

In summary, we found that there is a family of GVs in both study populations that follows the same direction of association, although the absolute values of the effects displayed in the NARAC study are more extreme than the ones from the EIRA study for both protective and risk GVs. We chose to use these GVs, with the aim of finding more specific effects from individual genetic variations for further optimization of analyses.

### 3.3. Candidate Combinations of Genotypes

We used all the available combinations from our genotyping data to differentiate GVs associated with disease. However, the functional consequences from these variations are likely to correspond only to a limited number of selected markers and most of the markers are not important for the association study. Therefore, for further studies it would be desirable to reduce the length of the GVs and to identify the most influential variants within GVs. By excluding unnecessary genetic variants from GVs, we could reconstruct shorter GVs, to facilitate inspection of the genotype content of the GVs in two contrast groups, chosen via two consequent statistical criteria.

In order to compare the results from the contrasting GVs of control and cases with different total numbers of individuals, we sum all IMs (see (4)) in each of categories 11 and 22 with the weights *n*_*ν*,*kk*_^*α*^/*N*_*α*_^sel^, where *n*_*ν*,*kk*_^*α*^ is number of individual cases (*α* = *s*, or “sick”) or controls (*α* = *h*, or “healthy”) in GV *ν*, belonging to the class *kk* = 11,22; here *N*_*α*_^sel^ are the numbers of individuals in the selected group *kk*, *N*_*α*,*kk*_^sel^ = ∑_*ν*∈*Ω*_*kk*_^*α*^_ *n*_*ν*,*kk*_^*α*^; notice that here the normalization is different from the one used in (7):

(21) Niγ,kkα=1Nα,kksel∑ν∈Ωkkαnν,kkαΨαXνiγΨα,      α=h,s; kk=11,22.

As expected, our data demonstrate strong association of RA with SE alleles (see Table 9). At the same time, the SNP rs1328674 does not show an association with RA, in contrast to a previous report [9], while we detected an association of the SNPs rs6314, rs977003, rs2070037, rs6313, and rs6311 in the EIRA study. Since not all of these results agree with previously published evaluation at this locus, we compare it with independent data from NARAC; see Table 10.

As can be seen, the data for NARAC fully confirm the conclusions derived from the EIRA data. The numbers for NARAC in Table 10 differ from those for EIRA (see Table 9) only slightly; therefore, the tendencies revealed in both cohorts from the same sets of GVs can be considered to be cross-validated.

Thus, selection of shorter GVs for statistical evaluation demonstrated how the overall analysis could be optimized: |*rs*2070037〉, |*rs*6313〉, |*rs*6311〉, and |SE〉 remain indicative for the difference between groups, whereas |*rs*6314〉, |*rs*977003〉, and |*rs*1328674〉 have no influence on association in the GVs of both EIRA and NARAC. These cropped GVs can be especially useful for optimizing the analysis of long GVs, since it can improve the statistical confidence of the results.

We identified significant heterogeneity of association of combinations of HTR2A variants, which depends on the absence or presence of a shared epitope allele in the GV. Several interesting observations have been found from application of the method to two RA study populations, EIRA and NARAC.

We found that the number of GVs that describe the study population for the chosen length of GV is relatively small: there are only 161 GVs required for description of the EIRA, with 2767 individuals, and 163 GVs for NARAC, consisting of 1974 people. A very high overlap between Swedish and North American study populations was detected in our analysis:* 131 of GVs are common to both study populations*.

Interestingly, some of the GVs contain only healthy controls and do not contain the case counterpart (subset of solely healthy persons), and* vice versa*; some of the GVs contain only cases and do not contain controls (solely ill). The statistical weight of these GVs, however, appeared to be small. Nevertheless, we have found similar behaviour in two independent cohorts and this observation deserves further investigation. Even after removing these “zero” GVs from consideration, the remaining set of common EIRA and NARAC GVs contains about 80% of the total number of individuals in each cohort.

Our literature search did not give much information about similar methods being used in genetic studies. The closest approach, haplotype analysis, is based on the classical genetic idea of transmission of the marker from parents to offspring and therefore the data in this analysis are arranged around the combination of genetic markers within a single chromosome. In an association study this approach may well serve in allelic mode, but it totally ignores the possible involvement of a second allele. In functional studies, selection of individuals by haplotypes is straightforward for homozygotic states but introduces multiple combinations in the case of heterozygosity and generates an extreme number of genotype groups in comparison with GVA. Experimental detection of haplotypes is a difficult procedure and the common statistical approach for assigning of haplotypes is never 100% unambiguous.

### 3.4. Genetic Vectors with Zero Values in One of the Subgroups, Experimental Data

In this section we illustrate previously defined “zero GV” detected in the EIRA and NARAC studies of RA. The number of individuals involved in each step of selection is decreased as shown in Table 11.

Thus, of 131 GVs common for EIRA and NARAC, 19 GVs happen to be zero GVs, that is, either *n*_*ν*,*α*_^*s*^ = 0 and *n*_*ν*,*α*_^*h*^ ≠ 0 or,* vice versa*, *n*_*ν*,*α*_^*s*^ ≠ 0 and *n*_*ν*,*α*_^*h*^ = 0. They contain only about 2% of individuals from the study populations (62/2727 in EIRA and 43/1938 in NARAC). It is interesting to compare the contents of GVs from the “solely sick” (SSGV) and the “solely healthy” (SHGV) subgroups. Since the number of individuals belonging to the classes of interest is small, it would be nice to have a look at the pooled data. The inspection, however, shows that the part of the GVs that belong to the zero GV subgroup in, say, EIRA, often does not belong to this subgroup in NARAC, and* vice versa*. These GVs lose their status of zero GVs in the pooled data.

The insufficient number of individuals in SSGV and SHGV subgroups does not make it possible to draw statistically confident conclusions if we work with each study population separately. We can, however, investigate whether there exists some cross-validated tendency, that is, the one observed in both EIRA and NARAC.

**Figure 7:**
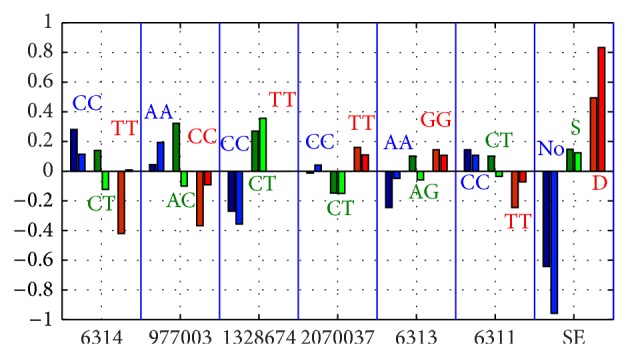
EIRA-versus-NARAC comparison charts for selected zero GVs: the differences in genotype frequencies *f*_*iγ*_^*s*^ − *f*_*iγ*_^*h*^ for GVs of “solely sick” and “solely healthy” individuals. Positive values indicate a propensity for disease, whereas the negative values suggest that a protective role is played by the corresponding genotype. In each genotype couple, the darker color on the left corresponds to EIRA, while the lighter color on the right presents the result for the NARAC difference of frequencies. S: single SE; D: double SE allele.

This can be visualized via GV frequency weighted index matrices for zero GVs. Since we are interested only in the groups of zero GVs, we will perform averaging over these subgroups only. In other words, (13) should be modified as follows:

(22) $$\begin{array}{c} \bar{C^{\text{sh}}}=\frac{1}{N_{\text{sh}}}\underset{\nu \subset \Omega_{0}^{\text{sh}}}{\sum }n_{\nu }^{\text{sh}}C_{v}^{\text{sh}},\\ \bar{C^{\text{ss}}}=\frac{1}{N_{\text{ss}}}\underset{\nu \subset \Omega_{0}^{\text{ss}}}{\sum }n_{\nu }^{\text{ss}}C_{v}^{\text{ss}}, \end{array}$$

where *n*_*ν*_^sh^ and *n*_*ν*_^ss^ are numbers of individuals in the GV *ν* belonging to the group SH or SS correspondingly; indices sh and ss mean “solely sick” and “solely healthy”; the numbers

(23) $$\begin{array}{c} N_{\text{sh}}=\underset{\nu \subset \Omega_{0}^{\text{sh}}}{\sum }n_{\nu }^{\text{sh}},\;\;N_{\text{ss}}=\underset{\nu \subset \Omega_{0}^{\text{ss}}}{\sum }n_{\nu }^{\text{ss}} \end{array}$$

are total numbers of individuals in the SSGV and SHGV subgroups and *C*_*v*_^sh^ and *C*_*v*_^ss^ are index matrices of the GV *ν* from SH and SS subgroups. Then we can compare EIRA and NARAC results for case-control differences of index matrices, $\bar{C^{\text{ss}}}-\bar{C^{\text{sh}}}$ for zero GVs. The result is shown in Figure 7.

Full coherence in EIRA and NARAC data display is only SE (no SE is protective, while double SE strongly associates with RA, which is expected) and the SNP rs1328674. The positive value of the difference for frequencies of the genotype (rs1328674,CT) means the tendency to disease, whereas the negative one for the genotype (rs1328674,CC) indicates the tendency to protect. All other genotypes do not display consistency between the results for EIRA and NARAC. Thus, although the fact of zero GVs existence is interesting, we have to admit that, due to the absence of (i) the consistency for two study populations and (ii) the possibility of drawing statistically confident conclusions, analysis of zero GVs should be taken with caution when the numbers of observations are too low. In practice, it will need replication study in very big cohorts.

## 4. Conclusions

### 4.1. On GVA

Our approach, GVA, is the method for genetic analysis based on testing of association to a complex trait simultaneously for combinations of variants (multiple markers)* inherent to individuals* without any assumptions about their statistical relations. It is based on ascribing to each person a multiple-spin quantum state, GV, with subsequent description of the study population as an ensemble of spin chains with a given (determined by experimental data) set of allowed states. The full data set is sorted into subsets, each of which is described by a single GV and the numbers of cases and controls representing this GV. We successfully tested this approach for the set of experimental data for RA and validated the previously known association with shared epitope alleles.

GVA has several advantages:

First, since GVA works with the genotype combinations as a single entity, without decoupling of random variables, the issue of possible statistical independence (as well as interaction, influence of the linkage disequilibrium, etc.) of separate genotypes does not affect analyses.

Second, being formulated for genotypes, it is free of the uncertainties connected with the use of an allelic model or haplotypes: it is, therefore, not necessary to guess a mode of dominance. At the same time, the frequencies of separate genotypes, or their pairs, triples, and so forth, can be easily expressed in terms of GV variables, which means that other analyses could be easily performed by simplification of the GV algorithm.

Third, from the perspective of functional genetics and personalized medicine, this approach provides the opportunity to directly assign individuals belonging to the risk group of interest to a genetically determined subset defined by GV. Since GVA includes in the analysis only the genotype (phenotype) combinations which are met in the population study, the number of combinations that need to be analyzed is significantly decreased compared to the number of random combinations of genetic markers; therefore, the volume of necessary calculations is greatly reduced. The problem of multiple combinations of haplotypes is also resolved in GVA.

The probability distribution for OR of each GV, which we derived from the hypergeometric distribution for the random variable “number of cases in GV,” happens to be asymmetric and is not Gaussian. For this reason, the standard method for evaluating the confidence accuracy could not be used. Thus, we instead evaluated the thresholds of the OR interval where OR fluctuations exceed 5% for each OR separately. The statistics of each GV depends on three parameters: the total number of cases, the total number of controls in the study population, and the total number of individuals (cases plus controls), described by the GV in question. Only those of the GVs for which OR falls outside of the interval of large OR fluctuations have been selected for further analysis. The dependence of the OR statistics for a particular GV on the number of individuals “belonging” to this GV creates the obvious disadvantage of GVA: with an increase of the GV length (the number of SNPs included in the GV) the total number of GVs describing the study population approaches the number of individuals, since each person has unique DNA and, therefore, statistical analysis becomes invalid.

Regarding optimization of GVA and reducing the number of markers in GV (cropping the vector size), we suggested using index matrices, uniquely characterizing each GV. By using this approach a comparative analysis of the “genotype content” can be done for the sets of GVs of interest, identifying the sets of genotypes that influence the difference between the control and case groups.

It is worth noting that GVA does not require any assumptions about statistical coupling between variables. The study of interaction and synergetic effects between particular SNPs can also be performed in terms of GVA. Indeed, the answer to the question whether pairs, or triples, of some of SNPs produce a much stronger association to the phenotype of interest than is expected from independent factors can be obtained from the analysis of the correlation functions between SNPs. Examples of this type of analysis have been discussed in [2].

### 4.2. On RA in EIRA and NARAC

Our analysis reveals that the majority of GV groups, 76.7% (or 81.4% of individuals in all GVs, Table 6), are consistent for the effect between EIRA and NARAC. The degree of inconsistence could be possibly explained by low number of observation and randomness, but also by influence of different environmental factors or by different genetic architecture of the locus (between included SNPs and at flanking regions).

We confirm the well-known fact that shared epitope (SE) is strongly associated with RA. What seems to be significant here is that the whole set of genotypes displays quite large differences in frequencies in the presence and absence of SE. The SNPs rs2070037, rs6313, and rs6311 seem to play a more important role in RA formation than they were considered to do in a previous study [9].

## Figures and Tables

**Table 1:** Structure of the study population in terms of GVs: each GV |*ν*_*i*_〉 is “populated” by *n*_*h*_ controls and *n*_*s*_ cases.

Type of GV	*n*_*h*_	*n*_*s*_	GV
*ν*_1_	*n*_*h*,1_	*n*_*s*,1_	|*ν*_1_〉
*ν*_2_	*n*_*h*,2_	*n*_*s*,2_	|*ν*_2_〉
⋮	⋮	⋮	⋮
*ν*_*N*_	*n*_*h*,*N*_GV__	*n*_*s*,*N*_GV__	|*ν*_*N*_GV__〉

**Table 2:** Encoding of SNP states: 1 = rs6314; 2 = rs977003; 3 = rs1328674; 4 = rs2070037; 5 = rs6313; 6 = rs6311. Each SNP can aquire any of genotype values listed in its column. Each GV is a product of SNP states in the order given in the table. Columns correspond to reference sequence number of SNP or to shared epitope (SE) genotype.

SNP	|1, *γ*_1_〉	|2, *γ*_2_〉	|3, *γ*_3_〉	|4, *γ*_4_〉	|5, *γ*_5_〉	|6, *γ*_6_〉	|SE, *γ*_7_〉
*γ*_*i*_ = 1	CC	AA	CC	CC	AA	CC	No
*γ*_*i*_ = 2	CT	AC	CT	CT	AG	CT	Single
*γ*_*i*_ = 3	TT	CC	TT	TT	GG	TT	Double

**Table 3:** Contingency table for a GV.

	Belongs to GV *ν*	Does not belong to GV *ν*
*s*	*n*_*ν*_^*s*^	*N*_*s*_ − *n*_*ν*_^*s*^
*h*	*n*_*ν*_^*h*^	*N*_*h*_ − *n*_*ν*_^*h*^

**Table 4:** The number of GVs in different groups: *N*_GV_^all^ is total number of GVs, *h* means “healthy controls,” *s* stands for cases (“sick”), and the index “with Zeros” denotes the GVs that do not contain individuals in one of the groups, *h* or *s*. These types of GVs are called zero GVs; “no Zeros” indicate that these sets of GVs do not contain zero GVs. Obviously, *N*_GV,noZeros_^*h*^ + *N*_GV,withZeros_^*h*^ = *N*_GV,noZeros_^*s*^ + *N*_GV,withZeros_^*s*^ = *N*_GV_^all^.

	*N*_GV_^all^	*N*_GV,noZeros_^*h*^	*N*_GV,noZeros_^*s*^	*N*_GV,withZeros_^*h*^	*N*_GV,withZeros_^*s*^	*N*_GV,noZeros_^Common^
GV_EIRA_	161	109	146	52	15	62
GV_NARAC_	163	135	101	28	62	62

**Table 5:** Numbers of vectors and individuals after separation of GVs specific only for one population and with zero frequency in any of subsets: *N*_GV,noZeros_^Common^ and *N*_GV_^allCommon^ are the numbers of common GVs in both sets, *n*_noZeros_^*α*^ is number of people after, and *n*_tot_^*α*^ is number of individuals before cleaning, index *α* = *h*, *s* (*E* ≡ EIRA, *N* ≡ NARAC, *h* means “healthy controls”, *s * stands for cases (“sick”)).

	*N*_GV,noZeros_^Common^	*N*_GV_^allCommon^	*n*_noZeros_^*h*^	*n*_tot_^*h*^	*n*_noZeros_^*s*^	*n*_tot_^*s*^	*n*_noZeros_^*h*+*s*^	*n*_tot_^*h*+*s*^
*E*	62	131	779 (83%)	938	1500 (83%)	1789	2279 (83%)	2727
*N*	62	131	866 (76%)	1134	711 (88%)	804	1577 (81%)	1938

**Table 6:** Consistent and inconsistent groups of GVs: Ω_11_ group with *N*_11_^GV^ = 31 represents GVs with increased risk in both cohorts, whereas Ω_22_ group with *N*_22_^GV^ = 15 represents GVs with decreased risk of RA. Ω_12_ and Ω_21_ groups represent inconsistent GVs and were excluded from further analyses.

		*N*_11_^*t*^	*N*_11_^*h*^	*N*_11_^*s*^			*N*_12_^*t*^	*N*_12_^*h*^	*N*_12_^*s*^
*N*_11_^GV^ = 31;	EIRA	1192	295	897	*N*_12_^GV^ = 6;	EIRA	147	42	105
	NARAC	822	296	526		NARAC	103	63	40

		*N*_21_^*t*^	*N*_21_^*h*^	*N*_21_^*s*^			*N*_22_^*t*^	*N*_22_^*h*^	*N*_22_^*s*^

*N*_21_^GV^ = 8;	EIRA	263	106	157	*N*_22_^GV^ = 15:	EIRA	670	334	336
	NARAC	205	91	114		NARAC	440	413	27

**Table 7:** Contrast GVs kk = 11 of individuals in strongest-risk group. Last row displays total number of individuals (the sum of corresponding columns). For briefness the notations are used: |*S*_1_〉 = |rs6314〉, |*S*_2_〉 = |rs977003〉, |*S*_3_〉 = |rs1328674〉, |*S*_4_〉 = |rs2070037〉, |*S*_5_〉 = |rs6313〉, |*S*_6_〉 = |rs6311〉, and |Υ〉 = |SE〉.

Cohorts						Genetic vectors						
EIRA			NARAC			|*S*_1_〉	|*S*_2_〉	|*S*_3_〉	|*S*_4_〉	|*S*_5_〉	|*S*_6_〉	|Υ〉
*n*_*ν*,11_^*t*^	*n*_*ν*,11_^*h*^	*n*_*ν*,11_^*s*^	*n*_*ν*,11_^*t*^	*n*_*ν*,11_^*h*^	*n*_*ν*,11_^*s*^	*γ*_1_	*γ*_2_	*γ*_3_	*γ*_4_	*γ*_5_	*γ*_6_	*γ*_7_
17	1	16	18	3	15	CC	CA	CC	TT	GG	CC	D
41	6	35	32	7	25	CC	AA	CC	TT	AG	CT	D
41	6	35	105	2	17	CC	AA	CC	CT	AG	CT	D
47	9	38	56	9	36	CC	CA	CC	TT	AG	CT	D
56	9	47	93	3	19	CC	AA	CC	CT	GG	CC	D
37	4	33	30	4	17	CC	CA	CC	CT	AG	CT	D
239	35	204	157	28	129	⇐∑‍						

**Table 8:** Contrast GVs kk = 22 of “most healthy” controls. Last row displays the sum for corresponding columns. For briefness the notations are used: |*S*_1_〉 = |rs6314〉, |*S*_2_〉 = |rs977003〉, |*S*_3_〉 = |rs1328674〉, |*S*_4_〉 = |rs2070037〉, |*S*_5_〉 = |rs6313〉, |*S*_6_〉 = |rs6311〉, and |Υ〉 = |SE〉.

EIRA			NARAC			|*S*_1_〉	|*S*_2_〉	|*S*_3_〉	|*S*_4_〉	|*S*_5_〉	|*S*_6_〉	|Υ〉
*n*_*ν*,22_^*t*^	*n*_*ν*,22_^*h*^	*n*_*ν*,22_^*s*^	*n*_*ν*,22_^*t*^	*n*_*ν*,22_^*h*^	*n*_*ν*,22_^*s*^	*γ*_1_	*γ*_2_	*γ*_3_	*γ*_4_	*γ*_5_	*γ*_6_	*γ*_7_
92	45	47	41	40	1	CC	AA	CC	TT	AG	CT	N
78	40	38	35	34	1	CC	AA	CC	CT	AG	CT	N
63	30	33	52	51	1	CC	CA	CC	CT	AG	CT	N
98	57	41	66	65	1	CC	CA	CC	TT	AG	CT	N
21	16	5	15	14	1	CT	CA	CC	TT	AG	CT	N
47	25	22	45	42	3	CC	CA	CC	TT	AA	TT	N
56	28	28	25	24	1	CC	AA	CC	TT	GG	CT	N
20	11	9	22	21	1	CC	AA	CC	TT	AA	TT	N
475	252	223	301	291	10	⇐∑‍						

**Table 9:** Averaged index matrices of contrast EIRA GVs: 11 = cases are followed by “#,” 22 = controls are followed by “∗,” and their difference is by “∗∗.” Strongest changes are marked by bold fonts. For briefness the notations are used: |*S*_1_〉 = |rs6314〉, |*S*_2_〉 = |rs977003〉, |*S*_3_〉 = |rs1328674〉, |*S*_4_〉 = |rs2070037〉, |*S*_5_〉 = |rs6313〉, |*S*_6_〉 = |rs6311〉, and |Υ〉 = |SE〉.

EIRA	|*S*_1_〉	|*S*_2_〉	|*S*_3_〉	|*S*_4_〉	|*S*_5_〉	|*S*_6_〉	|Υ〉
	*γ*_1_	*γ*_2_	*γ*_3_	*γ*_4_	*γ*_5_	*γ*_6_	*γ*_7_
	CC	AA	CC	CC	AA	CC	No
*γ*_*i*,*s*_ = 1	1^#^	0.5735^#^	1^#^	0^#^	0^#^	0.3088^#^	0^#^
*γ*_*i*,*h*_ = 1	0.9365^*^	0.4921^*^	1^*^	0^*^	0.1428^*^	0.1111^*^	1^*^
*γ*_*i*,*d*_ = 1	0.0635^**^	0.0815^**^	0^**^	0^**^	−0.1429^**^	0.1977^**^	−1^**^

	CT	AC	CT	CT	AG	CT	Yes
*γ*_*i*,*s*_ = 2	0^#^	0.4265^#^	0^#^	0.5637^#^	0.6912^#^	0.6912^#^	0^#^
*γ*_*i*,*h*_ = 2	0.0635^*^	0.5079^*^	0^*^	0.2778^*^	0.7460^*^	0.7460^*^	0^*^
*γ*_*i*,*d*_ = 2	−0.0635^**^	−0.0815^**^	0^**^	0.2859^**^	−0.0549^**^	−0.0549^**^	0^**^

	TT	CC	TT	TT	GG	DD	Double
*γ*_*i*,*s*_ = 3	0^#^	0^#^	0^#^	0.4363^#^	0.3088^#^	0^#^	1^#^
*γ*_*i*,*h*_ = 3	0^*^	0^*^	0^*^	0.7222^*^	0.1111^*^	0.1429^*^	0^*^
*γ*_*i*,*d*_ = 3	0^**^	0^**^	0^**^	−0.2859^**^	0.1977^**^	−0.1429^**^	1^**^

**Table 10:** Averaged index martices of contrast NARAC GVs: 11 = “sick” are followed by “#,” 22 = “healthy” are followed by “∗,” and their difference is followed by “∗∗.” Strongest changes are marked by bold fonts. For briefness the notations are used: |*S*_1_〉 = |rs6314〉, |*S*_2_〉 = |rs977003〉, |*S*_3_〉 = |rs1328674〉, |*S*_4_〉 = |rs2070037〉, |*S*_5_〉 = |rs6313〉, |*S*_6_〉 = |rs6311〉, and |Υ〉 = |SE〉.

NARAC	|*S*_1_〉	|*S*_2_〉	|*S*_3_〉	|*S*_4_〉	|*S*_5_〉	|*S*_6_〉	|SE〉
	*γ*_1_	*γ*_2_	*γ*_3_	*γ*_4_	*γ*_5_	*γ*_6_	*γ*_7_
	CC	AA	CC	CC	AA	CC	No
*γ*_*i*,*s*_ = 1	1^#^	0.4729^#^	1^#^	0^#^	0^#^	0.2636^#^	0^#^
*γ*_*i*,*h*_ = 1	0.9519^*^	0.4089^*^	1^*^	0^*^	0.2165^*^	0.0825^*^	1^*^
*γ*_*i*,*d*_ = 1	0.0481^**^	0.0639^**^	0^**^	0^**^	−0.2165^**^	0.1811^**^	−1^**^

	CT	AC	CT	CT	AG	CT	Yes
*γ*_*i*,*s*_ = 2	0^#^	0.5271^#^	0^#^	0.4109^#^	0.7364^#^	0.7364^#^	0^#^
*γ*_*i*,*h*_ = 2	0.0481^*^	0.5911^*^	0^*^	0.2921^*^	0.7010^*^	0.7010^*^	0^*^
*γ*_*i*,*d*_ = 2	−0.0481^**^	−0.0639^**^	0^**^	0.1188^**^	0.0354^**^	0.0354^**^	0^**^

	TT	CC	TT	TT	GG	TT	Double
*γ*_*i*,*s*_ = 3	0^#^	0^#^	0^#^	0.5891^#^	0.2636^#^	0^#^	1^#^
*γ*_*i*,*h*_ = 3	0^*^	0^*^	0^*^	0.7079^*^	0.0825^*^	0.2165^*^	0^*^
*γ*_*i*,*d*_ = 3	0^**^	0^**^	0^**^	−0.1188^**^	0.1811^**^	−0.2165^**^	1^**^

**Table 11:** Numbers of GVs and individuals in full cohorts, in common subsets of GVs, and in subsets of  “solely healthy” (SH, *n*_*ν*_^*s*^ = 0, *n*_*ν*_^*h*^ = *n*_*ν*_^*t*^) and “solely sick” (SS, *n*_*ν*_^*s*^ = *n*_*ν*_^*t*^, *n*_*ν*_^*h*^ = 0) individuals. The notations in the table are *ξ*_*t*_ = **N**_**t**_^**GV**^/*N*_*t*_, that is, (number of GVs)/(number of individuals); *ξ*_PS_ = **N**_**SS**_^**GV**^/*N*_SS_, that is, (number of SS GVs)/(number of SS individuals); *ξ*_SH_ = **N**_**SH**_^**GV**^/*N*_SH_, that is, (number of SH GVs)/(number of SH individuals); *N*_*s*_^tot^/*N*_*h*_^tot^ is (total number of cases)/(total number of controls); *ξ*_*t*_^*c*^ = **N**_**t**,**common**_^**GV**^/*N*_*t*_^*c*^, *N*_*s*_^*c*^/*N*_*h*_^*c*^ is (common number of cases)/(common number of controls); *ξ*_SS_^cr^ = **N**_**SS**,**cr**_^**GV**^/*N*_SS_^cr^, that is, (number of common SS GVs)/(number of common SS individuals); *ξ*_SH_^cr^ = **N**_**SH**,**cr**_^**GV**^/*N*_SH_^cr^, that is, (number of common SH GVs)/(number of common SH individuals).

		EIRA	NARAC
Full cohorts	*ξ*_*t*_	**161**/2767	**163**/1974
	*N*_*s*_^tot^/*N*_*h*_^tot^	1820/947	813/1161
	*ξ*_SS_	**52**/152	**28**/61
	*ξ*_SH_	**15**/21	**62**/263

GVs GVs common for EIRA and NARAC	*ξ*_*t*_^*c*^	**131**/2727	**131**/1938
	*N*_*s*_^*c*^/*N*_*h*_^*c*^	1789/938	804/1134
	*ξ*_SS_^cr^	**12**/55	**12**/31
	*ξ*_SH_^cr^	**7**/13	**7**/12

## List of Abbreviations

EIRA: Swedish Epidemiological Investigation of Rheumatoid Arthritis
GV: Genetic vector
GVA: Genetic vector approach
NARAC: North American Rheumatoid Arthritis Consortium
OR: Odds ratio
RA: Rheumatoid arthritis
SNP: Single-nucleotide polymorphism.
